# Overexpression of the rice AKT1 potassium channel affects potassium nutrition and rice drought tolerance

**DOI:** 10.1093/jxb/erw103

**Published:** 2016-03-11

**Authors:** Izhar Ahmad, Afaq Mian, Frans J. M. Maathuis

**Affiliations:** Department of Biology, University of York, York YO10 5DD, UK

**Keywords:** Drought, ion channel, *Oryza sativa*, OsAKT1, osmotic stress, rice.

## Abstract

Potassium (K^+^) is the most important cationic nutrient for all living organisms and has roles in most aspects of plant physiology. To assess the impact of one of the main K^+^ uptake components, the K^+^ inward rectifying channel AKT1, we characterized both loss of function and overexpression of OsAKT1 in rice. In many conditions, AKT1 expression correlated with K^+^ uptake and tissue K^+^ levels. No salinity-related growth phenotype was observed for either loss or gain of function mutants. However, a correlation between AKT1 expression and root Na^+^ when the external Na/K ratio was high suggests that there may be a role for AKT1 in Na^+^ uptake in such conditions. In contrast to findings with *Arabidopsis thaliana*, we did not detect any change in growth of AKT1 loss of function mutants in the presence of NH_4_^+^. Nevertheless, NH_4_^+^-dependent inhibition was detected during K^+^ uptake assays in loss of function and wild type plants, depending on pre-growth conditions. The most prominent result of OsAKT1 overexpression was a reduction in sensitivity to osmotic/drought stress in transgenic plants: the data suggest that AKT1 overexpression improved rice osmotic and drought stress tolerance by increasing tissue levels of K^+^, especially in the root.

## Highlight

Overexpression of the potassium channel OsAKT1 improves rice potassium nutrition and resilience to water stress.

## Introduction

Potassium (K^+^) is the primary cation in most plants. It affects all aspects of crop production including yield, resistance to pathogens and tolerance to abiotic stress such as salinity, lodging, and drought. Its uptake from the soil predominantly takes place via two separate mechanisms, one passive and one active (Maathuis and Sanders, 1996; Maathuis *et al.*, 1997; Véry and Sentenac, 2003; Rodríguez-Navarro and Rubio, 2006). Active K^+^ uptake is via H^+^-coupled carriers of the KUP/HAK family (Ahn *et al.*, 2004; Gierth *et al.*, 2005; Qi *et al.*, 2008) while the passive mechanism mainly consists of the K^+^-selective, Shaker type inward-rectifying channel AKT1 (Sentenac *et al.*, 1992; Hirsch *et al.*, 1998). AKT1 has been exhaustively characterized in heterologous systems and *in planta*, particularly in the model species *Arabidopsis thaliana* (Sentenac *et al.*, 1992; Hirsch *et al.*, 1998) but also in carrot and grapes (Formentin *et al.*, 2006; Cuéllar *et al.*, 2010), while a recent paper convincingly showed that OsAKT1 is orthologous to AtAKT1 (Li *et al.*, 2014). AKT1 channels are expressed predominantly, but not exclusively, in the roots and their activity is increased by membrane hyperpolarization with a threshold voltage of around −120 mV. Other factors such as pH, K^+^ and Ca^2+^ concentration impact on open probability. For example, the threshold membrane potential of AtAKT1 can shift to more negative values via heteromerization of AKT1 with KC1 subunits (Duby *et al.*, 2008; Geiger *et al.*, 2009). This lowers the affinity for K^+^ and is likely to prevent K^+^ leakage whenever the electrochemical K^+^ gradient is outwardly directed. Intriguingly, the same K^+^ deficiency also invokes post-translational modifications of the AKT1 protein that *increase* AKT1 activity. A signalling pathway that is believed to be initiated by Ca^2+^ promotes AKT1 open probability; it involves the calcineurin B-like proteins CBL1 and CBL9 and the CBL-interacting kinase CIPK23 (Xu *et al.*, 2006). The latter phosphorylates AKT1, which enhances channel activity and as such K^+^ uptake. This pathway has been shown to function in both Arabidopsis and rice (Xu *et al.*, 2006; Li *et al.*, 2014).

The large range of [K^+^]_ext_ where it is active makes AKT1 an ideal target to engineer increased K^+^ uptake in plants, especially when exposed to low K^+^ environments. This is important for several reasons. Firstly, while around 2.6% of the earth’s crust consists of K^+^, in most soil solutions K^+^ is too low (typically 10–100 µM) to attain maximum yield. In turn, this causes a large demand for K^+^ fertilization (usually as potash) for crop production but agricultural land around the globe is, or is becoming, deficient in potassium (Mengel *et al.*, 2001; Römheld and Kirkby, 2010) because K^+^ fertiliser costs are often high for farmers, especially in the developing world. K^+^ fertiliser production and transport also has significant negative impacts on the environment. Thus, developing plants that can support growth at lower ambient K^+^ is a sustainable way to reduce dependence on K^+^ fertilization. Secondly, increased K^+^ uptake is also likely to contribute to plant abiotic stress tolerance, particularly drought and salinity, even in K^+^-replete conditions. High tissue K^+^ levels also promote nitrogen use efficiency and can reduce the toxic effects of other nutrients such as ammonium (NH_4_^+^) (Wang *et al.*, 1996).

We set out to characterize the function of AKT1 in rice by studying both loss and gain of function in OsAKT1. As was shown recently (Li *et al.*, 2014), loss of function reduced tissue K^+^ and reduced growth on low-K^+^ media. However, ectopic OsAKT1 expression led to augmented K^+^ uptake and, relative to wild type plants, better growth at low ambient K^+^ concentrations and in the presence of water stress.

## Materials and methods

### Plant materials and growth

Rice (*Oryza sativa* L.) subgroup *Japonica* cv. Nipponbare and cv. Dongjin seeds were geminated on terra green and kept for 5 days in the dark at 28 °C and 90% relative humidity. The germinated seedlings were transferred to plastic boxes containing 2 litre of growth medium each. The standard growth medium consists of macronutrients (2.9mM NH_4_NO_3_, 0.3mM NaH_2_PO_4_, 0.5mM K_2_SO_4_, 1mM CaCl_2_, 1.6mM MgSO_4⋅_7H_2_O), micronutrients (Yoshida *et al.*, 1976) and Na_2_SiO_3_ (0.18 g l^–1^). K_2_SO_4_ in the standard medium was replaced with an equimolar quantity of Na_2_SO_4_ for the ‘0 K’ condition. The standard medium was supplemented with additional KCl and NaCl to increase the concentration of Na^+^ and K^+^ up to 60 or 75mM in the medium for the salt-stress treatments, while for osmotic-stress treatments it was supplemented with 5%, 10% or 15% polyethylene glycol (PEG)-4000. Solutions were prepared with deionized water and pH was adjusted to 5.6–5.7.

Glass house conditions were: 16h light/8h dark; 28/24 °C day/night; 60% relative humidity with light radiation of about 160W m^–2^. The growth medium was changed every 3 days. Seedlings were grown in standard medium for 3 weeks before treatments were applied, which lasted for 2 weeks unless otherwise indicated.

To test the effect of ammonium, plants were grown in media where N was provided exclusively in the form of NO_3_^–^ or NH_4_^+^. The impact of drought was measured by transferring 4-week-old plants to pots containing soil (John Innes No. 2 Compost + perlite 2*–*5mm). Non-treated plants were watered twice per week to 100% field capacity while drought stress was applied by watering plants to approximately 40% field capacity. After 6 weeks’ treatment, the fresh weight of the plants was recorded and relative growth rate (RGR) was determined. In all cases, RGR was calculated according to Poorter & Garnier (1996).

### Transposon and T-DNA insertion lines for OsAKT1

Two putative insertion lines of *AKT1* were used in this study to analyse the disruption of this gene in rice under different conditions. The putative transposon insertion line T14884T in the Nipponbare background was obtained from the Rice Genome Resource Centre of the National Institute of Agrobiological Sciences (RGRC-NIAS), Japan. The seeds for the Postech T-DNA insertion line PFG_1B-16021 in the Dongjing background were obtained from the Crop Biotech Institute, Department of Plant Systems Biotech, Kyung Hee University, Republic of Korea. The transposon insertion was in the 8^th^ exon of the T14884T line while the T-DNA is inserted in the 5′UTR region, approx 70bp upstream of the start codon of line PFG_1B-16021.

### Analysis of loss of function mutants:

The rice *akt1* transposon insertion line T14884T and T-DNA insertion line PFG_1B-16021 were characterized by PCR and RT-PCR using different sets of primers. Line T14484T was analysed with transposon primer 5′-AGGTTGCAAGTTAGTTAAGA-3′ and a gene-specific reverse primer 5′-ACGTAGCGAATCCATAAGCTCC-3′ to validate the presence of the transposon (Supplementary Fig. S1 at *JXB* online). Homozygosity of the insertion was confirmed with gene-specific forward (5′-ACCAACATGGCTTGTTCTTGAC-3′) and reverse (5′-TGAAGACCTTCTGAATCTGTC-3′) primers spanning the transposon insertion site (Supplementary Fig. S1). The PFG_1B-16021 line was previously characterized by Li *et al.* (2014). Amplification of 1B-16021 DNA with gene-specific forward (5′-TTTAAGACAAACCCAGACAGC-3′) and reverse (5′-CCAGTATAGCAGGACTGTACAC-3′) primers showed homozygous presence of T-DNA. Both lines were subsequently analysed by RT-PCR on root-derived cDNA with gene-specific forward (5′-TCGACAAGCAGGACGGCAA-3′) and reverse (5′-CAGTTATTCCTTAGCTAACCGTT-3′) primers. Throughout the text, line T14884T and line PFG_1B-16021 are referred to as *akt1-1* and *akt1-2* respectively.

### Rice transformation

The full length OsAKT1 open reading frame (ORF) was amplified with Phusion Hot Start DNA polymerase (New England Biolabs, UK) using AKT1 cDNA (accession AK120308) as template and primers corresponding to the 5′ and 3′ ends of OsAKT1 with added *Hin*d3 and *Eco*R1 restriction sites (5′-CGGGATCCGGCATGGGGCTCGATTT-3′ and 5′-GAACGAGATTAATTTACAGA-3′, respectively). The OsAKT1 ORF was then ligated into the corresponding sites of the pART27 plasmid downstream of the CaMv-35S promoter. The promoter-AKT1 cassette was restricted with *Eco*RV and inserted into the binary vector pGreen (Vain *et al.*, 2004). Subsequently, pGreen and pSoup were introduced into the Agrobacterium strain AGL1 to transform rice calli as described by Vain *et al.* (2004).

### Validation and evaluation of AKT1-OX transgenic rice

Two sets of primers were used to confirm the presence of the transgene in two putative AKT1-OX lines. Hygromycin-specific forward (5′-GGATATGTCCTGCGGGTAAA-3′) and reverse (5′-ATTTGTGTACGCCCGACAG-3′) primers, 35S promoter forward (5′-AAACCTCCTCGGATTCCATT-3′) and *AKT1* gene-specific reverse primer (5′-ACGTAGCGAATCCATAAGCTCC-3′). Hygromycin-resistant primary transformants that segregate 3:1 in the T1 were selfed and homozygous lines of AKT1 were identified in the T3 generation. The total RNA from leaf tissues of WT and OX lines was extracted and cDNA synthesized. qRT-PCR was carried out using an ABI 7300 analyser and the AKT1-specific forward and reverse primers 5′-AGAGATCCTTGATTCACTGCC-3′ and 5′-TCTACTAACTCCACACTACCAG-3′. The quantitative analyses were carried out in triplicate using SYBR Green master mix and rice Histone3 (Os06g0130900) to normalize the data (Supplementary Fig. S2). The progenies of the selfed heterozygous transgenic plants that lacked the transgene in the T3 generation were identified and used as control lines. These are denoted as ‘control’ or wild type (WT) plants.

### Ion content analyses in root and shoot tissues

K^+^ and Na^+^ contents of roots and shoots were measured using flame photometry (Sherwood flame photometer-410, Cambridge, UK). Plants were separated into roots and shoots and roots were washed with 20mM LaCl_3_ solution for 10min. Samples were dried at 80 °C for 3 days and extracted for 24h with 10ml of 20mM LaCl_3_.

### K^+^ depletion assays

Four-week-old plants (*n*=3), grown in ammonium-free or ammonium-containing conditions, were exposed to 50ml medium containing 50 µM K^+^ and ammonium at the indicated levels. Medium samples were taken at regular intervals to determine changes in medium K^+^. Loss of volume was corrected by addition of K^+^-free medium. K^+^ uptake/efflux was normalized to total root FW.

### Membrane potential recordings

Plants were grown as described above. Intact (4–5cm) roots from 3- to 4-week-old plants were fixed on a Plexiglas stage. Roots were immersed in assay buffer (0.2mM CaCl_2_, 1mM MES, pH 6). The K^+^ level was adjusted to 0, 0.1 and 1mM using KCl. Individual cells were impaled with 0.2M KCl-filled glass pipettes. During impalement, the bath solution was continuously refreshed at a rate of ~1mL min^–1^. Data are the average of at least four impalements in two different plants for each K^+^ level.

### Whole-leaf conductance measurements

Intact leaves from 4-week-old plants were used to measure leaf conductance and rate of photosynthesis by using an infrared gas analyser, Li-Cor 6400 (LI-COR, Cambridge, UK). For each genotype, three leaves (second, third and fourth) per plant (at six-leaf stage) were used and these were derived from three separate plants (*n*=9).

### Statistical treatment

In all cases, data are from at least three independent experiments and the error bars in the figure represent the standard errors. An asterisk denotes a significant difference by *t*-test at a probability level of *P*<0.05 between the wild type and other genotypes.

## Results

### AKT1 overexpression improves K^+^ nutrition

**Fig. 1.**
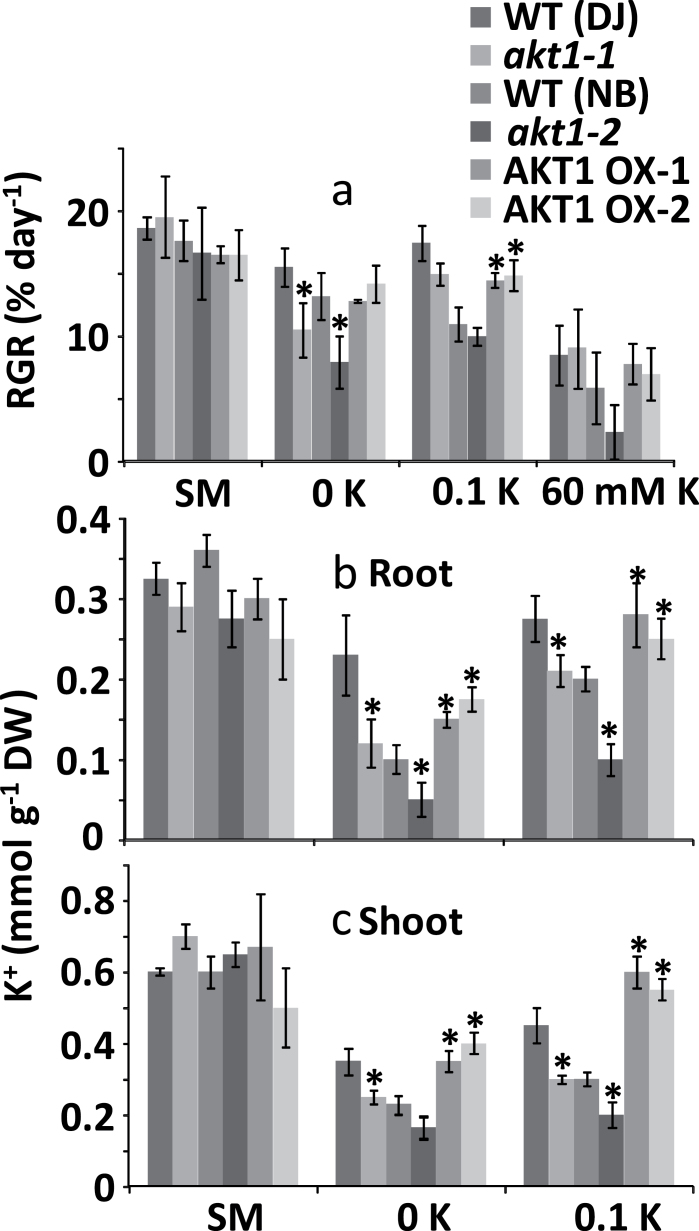
Relative growth rate and tissue K^+^ for hydroponically grown rice. (a) Relative growth rate (RGR, % day^−1^) of 5-week-old rice plants exposed to standard medium (SM), and media containing 0mM K^+^, 100 µM K^+^, and 60mM K^+^. (b) Root K^+^ content of plants grown in SM, 0mM K^+^ and 100 µM K^+^ conditions. (c) Shoot K^+^ content of plants grown in the conditions mentioned under (b). *Significant difference by *t*-test at a probability level of *P*<0.05 between each genotype and its respective wild type.

In standard conditions (1mM K^+^) no significant differences were observed in relative growth rate (RGR) between loss of function (KO), wild type (WT) and overexpressor (OX) genotypes (Fig 1a). Similarly, Fig 1a depicts a lack of significant differences in RGR when plants were grown at high K^+^ (60mM). In contrast, when plants were grown at 0K (i.e. no K^+^ added, equivalent to 2–3 µM) the KO lines showed less growth than WT plants while growth of AKT1-OX plants was not affected. In these conditions it is unlikely that AKT1 participates in K^+^ uptake. However, loss of AKT1 may reduce Na^+^ uptake (see below) and since Na^+^ is beneficial in low K^+^ conditions (Amtmann and Sanders, 1999; Amtmann and Beilby, 2010; Blumwald, 2000; Buschmann *et al.*, 2000; Maathuis, 2007) such reduction could negatively impact on growth at low ambient K^+^.

When plants were grown on a 100 µM K^+^ medium, AKT1 null mutants grew slightly worse than WT plants but this trend was only significant for *akt1-1* and not *akt1-2*. Presumably, at this intermediary concentration HAK-type transporters can largely compensate for the loss of AKT1. In contrast to KO lines, OX lines did significantly better than WT or KO mutants at this level of ambient K^+^ with approximately 20% and 25% higher RGR, respectively. To assess whether AKT1 can mediate K^+^ uptake in these conditions, the membrane potential of epidermal root cells was determined. Table 1 shows an average potential of −142 mV with 0.1mM external K^+^. The potassium potential (*E*_K_) in these conditions is only slightly more positive (around −130 to −140 mV), suggesting that AKT1-mediated fluxes will be small but, over prolonged periods, could nevertheless generate a sizeable contribution.

**Table 1.** Rice root membrane potentials Membrane potentials (*E*_m_) were recorded from epidermal rice root cells bathed in 0.2mM CaCl_2_, pH 6 buffer (see Methods). Plants were K-starved for 5 d in 0K medium. *E*_K_ was calculated using K^+^ tissue data from Fig. 1. Data are given ±SD and the number in parentheses denotes the number of impalements. n.d.: not defined.

	Buffer [K^+^]		
*E*	0 mM	0.1 mM	1 mM
*E*_m_ (mV)	−154±17.7 (5)	−142±18.4 (4)	−109±15.2 (4)
*E*_K_ (mV)	n.d.	−129 to −138	−84 to −96

The differences in growth rates between genotypes may be directly related to changes in tissue levels of K^+^; the low K^+^ (0 or 100 µM) media did cause a drop in tissue [K^+^] of all genotypes (Fig. 1b, c) but relative to standard medium the reduction in KO lines was more severe than that in OX lines. For example, in the 100 µM medium root and shoot K^+^ of WT plants decreased by 10–20% (roots) and 30–40% (shoots) while in the KO plants this was 40–70% (roots) and 50–60% (shoots). In contrast, there was hardly any change in K^+^ levels of the OX lines.

**Fig. 2.**
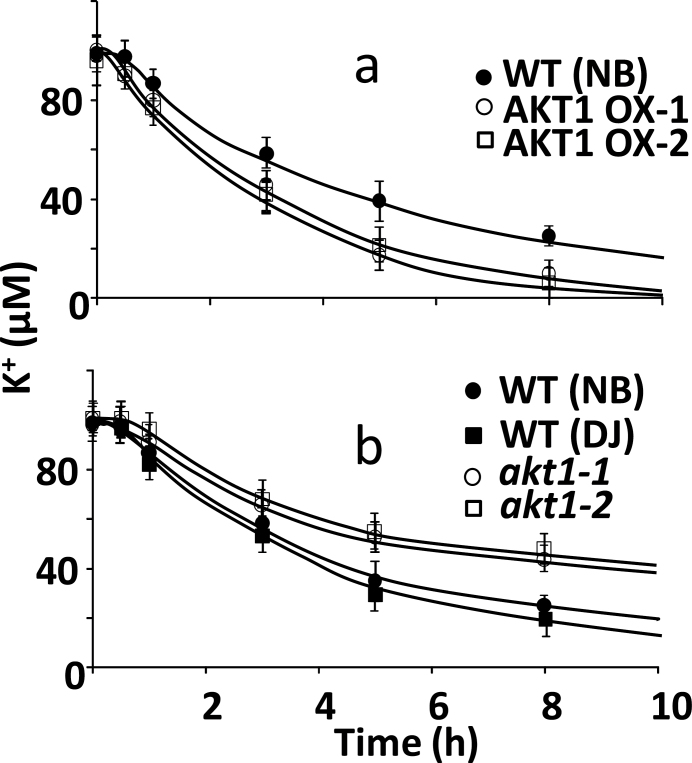
Net K^+^ uptake in hydroponically grown rice. (a) Net K^+^ uptake for WT (Nipponbare) and two independent AKT1 overexpressing lines from a solution containing 100 µM K^+^. (b) Same as (a) for WT (NN and DJ) and *akt1* loss of function mutants.

To assess if the altered tissue K^+^ concentration in the KO and OX plants was due to changes in K^+^ uptake, depletion experiments at 100 µM external K^+^ were carried out to measure net K^+^ uptake (Fig. 2). Our results show that OsAKT1 expression levels correlated with uptake, with higher net K^+^ uptake in the OX lines and slower K^+^ depletion in the KO lines compared with WT. This supports the notion that in this range of ambient K^+^ AKT1 is a major contributor to K^+^ uptake and AKT1 overexpression endows OX plants with the capacity to maintain higher K^+^ tissue levels.

### AKT1 does not affect NH_4_^+^ toxicity in rice

**Fig. 3.**
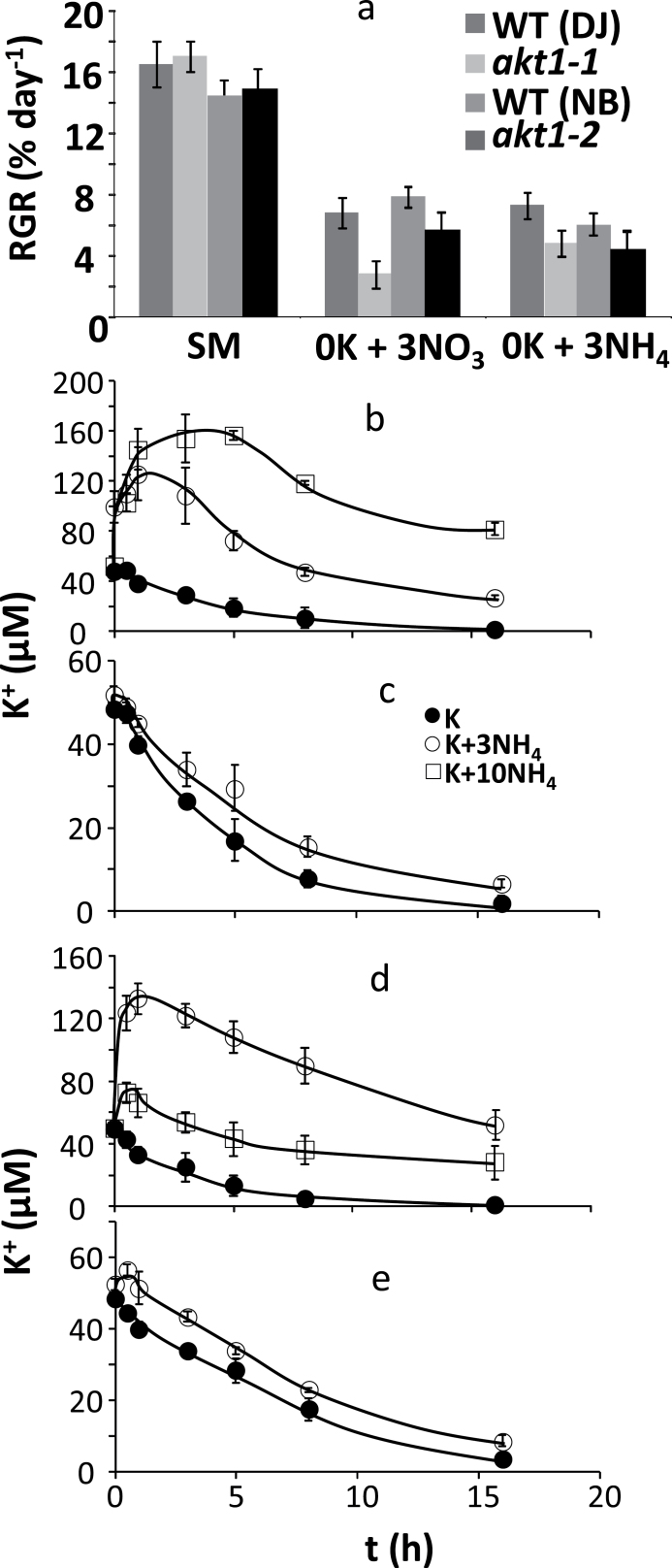
The effect of ammonium on growth rate and K^+^ uptake. (a) Relative growth rate (RGR, % day^−1^) of 5-week-old rice plants exposed to standard medium (SM), and media containing 0mM K^+^ and zero ammonium (0K + 3NO_3_), or 0mM K^+^ plus 3 or 10mM ammonium (0K + 3NH_4_ or 0K + 10NH_4_). (b–e) Net K^+^ uptake for WT (Nipponbare; b and c) and *akt1-2* (d and e) plants from a solution containing 50 µM K^+^, either in the absence or the presence of 3 or 10mM NH_4_^+^. Plants were pregrown in the absence of NH_4_^+^ (b and d) or with 1.5mM NH_4_^+^ in the growth medium (c and e). *Significant difference by *t*-test at a probability level of *P*<0.05 between each genotype and its respective wild type.

Previous work with Arabidopsis showed that growth in the absence of AKT1 is reduced on low K^+^ media only in the presence of NH_4_^+^ because the remaining K^+^ transporter (of the HAK/KUP family) is inhibited by this cation (Hirsch *et al.*, 1998). We therefore tested whether a similar growth effect occurs in rice by measuring growth of plants exposed to low [K^+^]_ext_ with and without NH_4_^+^. We exposed WT (DJ and NB) and the *akt1-1* and *akt1-2* null mutants to a 0K, 0 NH_4_ medium (where N is exclusively offered in the form of NO_3_^–^) and the same medium with N exclusively in the form of NH_4_^+^. Comparison of the 0K+3NO_3_ and 0K+3NH_4_ data in Fig. 3 shows that growth rates are similar, irrespective of the presence of NH_4_^+^, for either WT or KO genotypes. Thus, in contrast to what was found with Arabidopsis (Hirsch *et al.*, 1998) the presence of NH_4_^+^ does not cause an inhibitory effect on growth of *akt1* KO mutants in rice. To further test the interaction between K^+^ and NH_4_^+^ in rice, we measured the effect of NH_4_^+^ on net K^+^ uptake in WT (DJ) and *akt1-1* mutants by following K^+^ depletion from a medium with 0.05mM K^+^ and different levels of NH_4_^+^. Figure 3b shows that, when plants are pregrown in the absence of NH_4_^+^, the presence of 3mM NH_4_^+^ in the uptake buffer leads to a large K^+^ efflux. This effect is even more pronounced when the NH_4_^+^ concentration is raised to 10mM. However, when plants are grown in standard medium which contains approx 1.5mM NH_4_^+^ and NO_3_^–^ (see Methods), the presence of 3mM NH_4_^+^ is negligible (Fig. 3c). A very similar pattern was obtained with *akt1-1* mutants (Fig. 3d, e). In all, these findings suggest the presence of a few millimolar NH_4_^+^ does not negatively affect rice growth or net K^+^ uptake, even when measured in an AKT1 null mutant, as long as plants are accommodated to the presence of NH_4_^+^ in the growth medium.

### AKT1 expression affects rice growth during water stress

**Fig. 4.**
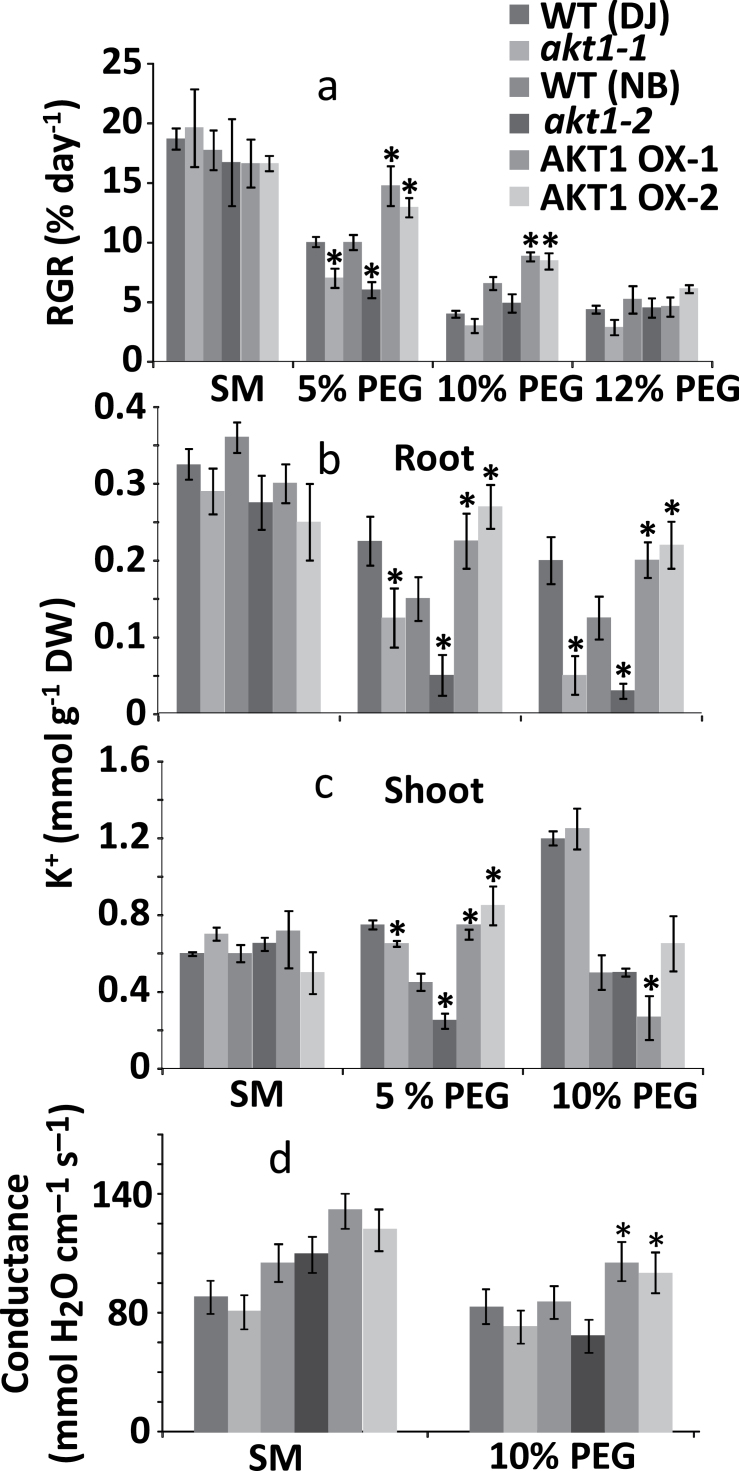
Relative growth rate and K^+^ content of osmotically stressed rice. (a) Relative growth rate (RGR, % day^−1^) of 5-week-old rice plants exposed to standard medium (SM), and media containing 5, 10 or 12% polyethylene glycol (PEG). (b) Root K^+^ content of plants grown in the standard medium, and 5 and 10% PEG conditions. (c) Shoot K^+^ content of plants grown in the conditions mentioned under (b). (d) Leaf stomatal conductance of the five genotypes for plants grown in standard medium or plants exposed to osmotic stress. *Significant difference by *t*-test at a probability level of *P*<0.05 between each genotype and its respective wild type.

To further assess how OsAKT1 expression affects rice growth, we grew WT, KO and OX lines hydroponically in control medium or media with PEG (5, 10 and 12%) to create osmotic stress. Figure 4a shows that during growth in PEG, KO lines tended to grow worse than the WT, although this was only significantly so with 5% PEG. OX lines showed an opposite trend with significantly improved growth relative to WT plants when plants were exposed to 5 or 10% PEG. The improved water-stress tolerance of AKT1-OX plants may be due to increased K^+^ accumulation in root or shoot tissues. We therefore determined tissue [K^+^] for the conditions where a significant growth difference was observed (5 and 10% PEG). At 10% PEG, KO lines had extremely low (30–50 µmol g^−1^ DW) K^+^ contents (Fig. 3b) while both OX lines contained significantly more K^+^ in their root tissue than WT. In general, the root K^+^ levels tended to mirror growth characteristics, with KO plants containing less and OX plants containing more K^+^. For shoots, a similar pattern was obtained in the 5% PEG condition but not in the 10% PEG medium where OX1 showed an actual decrease in K^+^ content (Fig. 4c).

Improved growth during osmotic stress could be due to increased tissue K^+^ levels but AKT1 could also participate in stomatal regulation (e.g. Nieves-Cordones *et al.*, 2012). Stomatal conductance of mature leaves did not differ significantly when plants were grown on standard medium (Fig. 4d) but when exposed to osmotic stress (10% PEG), OX lines showed increased stomatal conductance. This did not lead to any differences in tissue water content when either KO or OX lines were compared with WT. However, there was a significantly higher water content in OX lines when compared with KO lines (Supplementary Fig. S1).

**Fig. 5.**
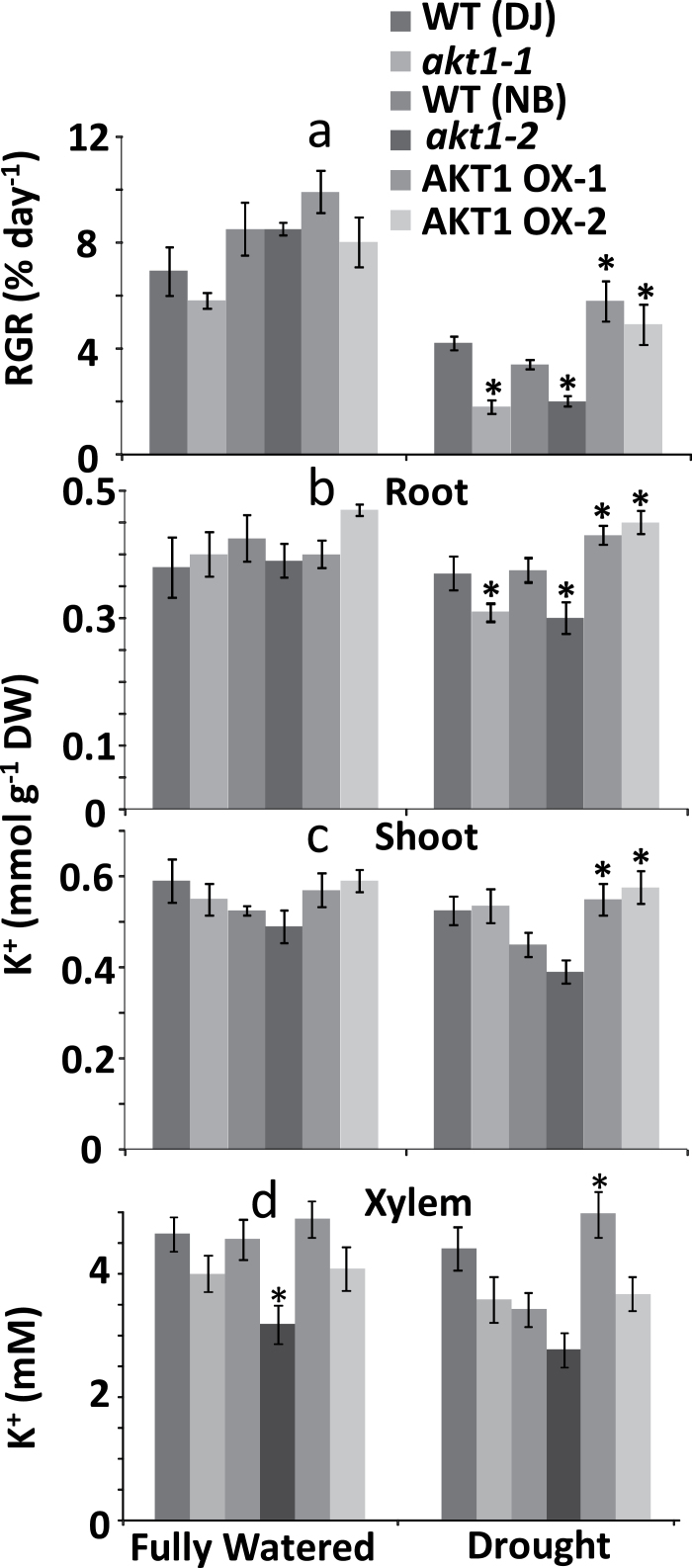
Growth and tissue and xylem K^+^ content of drought treated rice. (a) RGR of pot grown plants exposed for 6 weeks to full watering (100% field capacity) or limited water supply (~40% field capacity). (b) Root K^+^ content of plants grown in the conditions mentioned under (a). (c) Shoot K^+^ content of plants grown in the conditions mentioned under (a). (d) Xylem sap K^+^ concentration. *Significant difference by *t*-test at a probability level of *P*<0.05 between each genotype and its respective wild type.

We further investigated the water-stress phenotype by growing mature plants of all three genotypes in soil, applying full watering (100% field capacity) or drought stress (40% field capacity) for a period of 6 weeks. Figure 5a shows that in soil too, KO lines do worse and OX lines do better than their respective WT. Figure 5b shows that after drought exposure, plants with the KO genotype contained around 15% less K^+^ in their roots while OX plants accumulated around 15% extra K^+^. Leaf tissues of the OX genotype contained around 15–20% more K^+^ than WT shoots (Fig. 5c). The trends observed in tissue K^+^ levels were also reflected in xylem sap K^+^ levels (Fig. 5d). The xylem sap of KO plants showed lower K^+^ concentrations albeit not statistically significant, and higher in OX lines compared with control plants.

### AKT1 does not impact on salt tolerance

**Fig. 6.**
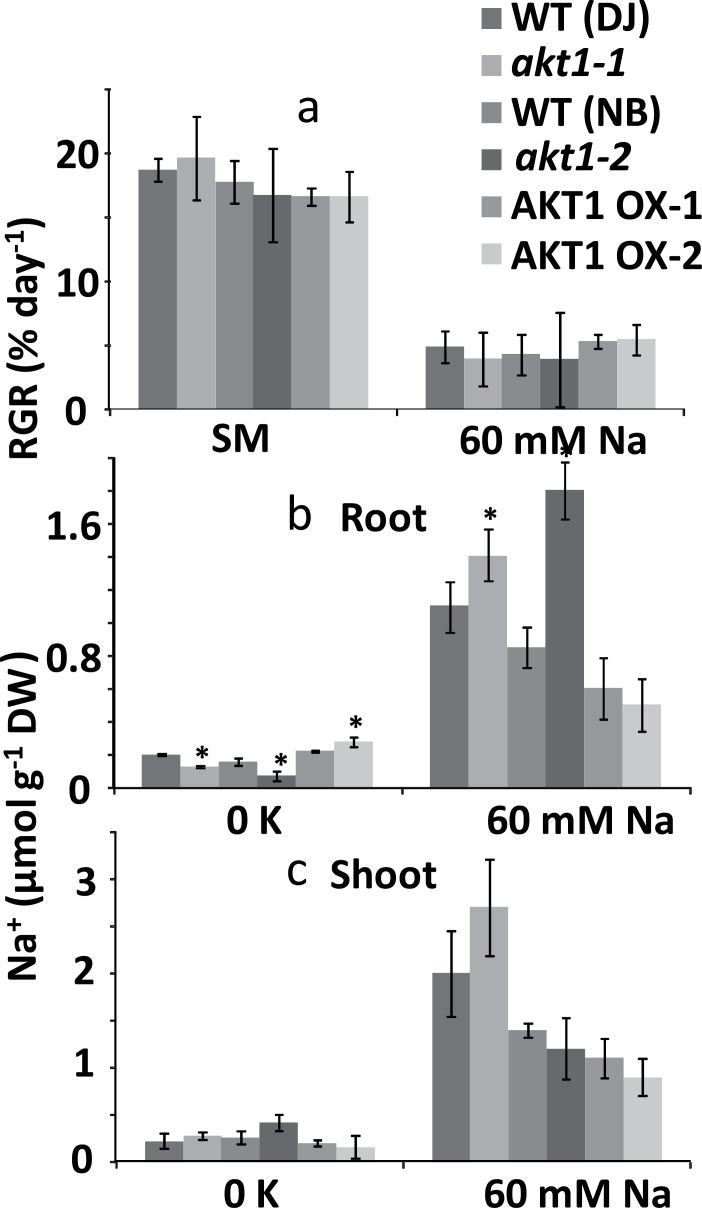
Relative growth rate and tissue Na^+^ for hydroponically grown rice. (a) Relative growth rate (RGR, % day^−1^) of 5-week-old rice plants exposed to standard medium (SM), and 60mM Na^+^. (b) Root Na^+^ content of plants grown in 0K and 60mM NaCl conditions. (c) Shoot Na^+^ content of plants grown in 0K and 60mM NaCl conditions. *Significant difference by *t*-test at a probability level of *P*<0.05 between each genotype and its respective wild type.

It has been suggested that OsAKT1 is involved in rice salt tolerance for several reasons: (i) AKT1-mediated K^+^ inward current was shown to be considerably diminished after growth in the presence of 150mM NaCl (Fuchs *et al.*, 2005), (ii) salt affects AKT1 transcript level in different ways for salt-sensitive and salt-tolerant rice varieties (Golldack *et al.*, 2003), and (iii) pharmacological profiles hinted at Na^+^ uptake through AKT1 (Anil. *et al.*, 2005; Wang *et al.*, 2007). Because 150mM is lethal within days for most rice cultivars, we used more moderate NaCl concentrations of 60 (Fig. 6) and 75mM (data not shown) to test growth in saline conditions. Figure 6a shows there is no difference in RGR between genotypes at 60mM NaCl and similar results were obtained in the presence of 75mM salt stress. Tissue K^+^ levels of salt grown plants were similar (Supplementary Fig. S2) but interestingly, growth in 60mM NaCl led to a higher root [Na^+^] for both the KO lines while it showed a, non-significant, downward trend in OX plants (Fig. 6b, right panel). No significant differences in leaf [Na^+^] were recorded (Fig. 6c, right panel). Thus, the pattern of Na^+^ accumulation in the roots is the reverse of what would be expected if AKT1 mediated Na^+^ uptake, and therefore argues against a role of this channel during salt stress. In contrast, growth in the 0K condition (which contains ~1mM NaCl, a level that is beneficial to the plant) showed changes in AKT1 expression and root Na^+^ that correlated, i.e. reduced root Na^+^ in both KO lines and increased Na^+^ in OX2 (Fig. 6b, left panel).

## Discussion

K^+^ is essential for a number of biochemical and biophysical functions in plant cells (Ahmad and Maathuis, 2014; Zörb *et al.*, 2014) and its cytoplasmic concentration is believed to be tightly controlled (Walker *et al.*, 1996; Halperin and Lynch, 2003; Kronzucker *et al.*, 2003). To maintain adequate K^+^ levels, plants have a number of K^+^ uptake systems of which AKT1 is one of the major players. Originally characterized in Arabidopsis, AKT1 is a voltage-dependent inward rectifying channel with a high selectivity for K^+^, expressed mainly in the root cortex (Lagarde *et al.*, 1996). Loss of function mutations in AtAKT1 (Hirsch *et al.*, 1998) have shown that this channel mediates K^+^ uptake over a large range of external K^+^ concentrations. Furthermore, expression data (Golldack *et al.*, 2003), pharmacology (Anil *et al.*, 2007; Wang *et al.*, 2007) and electrophysiological characterization (Fuchs *et al.*, 2005) suggested that OsAKT1 may also play a role in salt tolerance.

Previously, Li *et al.* (2014) showed that loss of function in OsAKT1 leads to reduced net K^+^ uptake and impaired growth in rice. However, the study by Li *et al.* did not extensively report on phenotypic characteristics of rice *akt1* mutants, nor did it investigate *AKT1* gain of function mutants.

### OsAKT1 impacts on K^+^ nutrition

Soils that are K^+^ deficient are becoming increasingly common and K^+^ fertilization is a considerable cost to farming. Enhanced K^+^ uptake capability, especially at low [K^+^]_ext_, could help access K^+^ stores that are otherwise not available to the plant and thus stimulate growth when ambient K^+^ is low. When grown in severe K^+^-deficient conditions (0K), AKT1 overexpression did not show an effect on rice growth (Fig. 1), but in the KO lines RGR was reduced as were root and shoot K^+^ levels. In these conditions the relative contribution of AKT1 to K^+^ uptake is non-existent because of the millimolar affinity of K^+^ inward rectifying channels (e.g. Maathuis and Sanders, 1995) and the requirement for a membrane potential that is more negative than the K^+^ Nernst potential. The latter is rarely met when [K^+^]_ext_ is less than around 20 µM (Maathuis and Sanders 1993; Nieves-Cordones *et al.*, 2008; Geiger *et al.*, 2009). Thus, the observed phenotype of the KO lines in the 0K condition is most likely due to functions of OsAKT1 that are not directly related to K^+^ uptake. OsAKT1 is expressed in almost all rice tissues and organs (Genevestigator) and, for example, alteration in guard cell functioning or tissue K^+^ distribution could cause the observed changes in growth. Alternatively, loss of function in OsAKT1 could lead to changes in expression of other transporters such as high-affinity K^+^ uptake systems in the KUP/HAK family (e.g. Nieves-Cordones *et al.*, 2010).

At an ambient K^+^ concentration of 100 µM, AKT1 is likely to participate in K^+^ uptake (Hirsch *et al.*, 1998; Spalding *et al.*, 1999; Li *et al.*, 2014). KO lines showed slightly less growth with this K^+^ regime but, in contrast to results from Li *et al*., the reduction was not significant. A possible reason for this divergence may be the studied material, which is seedlings in the work of Li *et al*. whereas data reported here are from mature plants. At 0.1mM, OX lines significantly outgrew WT plants and showed higher tissue K^+^ levels, suggesting that extra K^+^ uptake may be involved. With 0.1 and 30mM values for [K^+^]_ext_ and tissue [K^+^], respectively, a membrane potential of approximately −140 mV would suffice for channel-mediated K^+^ uptake.

The altered uptake and tissue level of K^+^ in KO and OX lines could impact on K^+^ use efficiency (KUE; yield or growth per unit of K^+^ input). Using this definition and the shoot K^+^ levels measured at 100 µM external K^+^ (Fig 1), KUE for the WTs (DJ and NB) was 0.33 and 0.32% RGR day^−1^ µmol K^−1^; for the KO lines (*akt1-1* and *akt1-2*), 0.40 and 0.43; and for OX1 and OX2, 0.29 and 0.30. Thus at moderately low K^+^, overexpression of AKT1 improves growth but at the cost of a reduced KUE. Indeed, KUE was highest in the KO lines due to the relatively large reduction in shoot [K^+^] of this genotype.

### Loss of function in AKT1 did not alter NH_4_^+^ sensitivity

*Arabidopsis akt1* KO lines only show a clear phenotype at low K^+^ in the presence of millimolar levels of ammonium (NH_4_^+^) (Hirsch *et al.*, 1998). We did not find evidence of increased NH_4_^+^ toxicity in the rice AKT1 loss of function mutants (Fig. 3). Similarly, net K^+^ uptake was only slightly affected by 3mM NH_4_^+^ provided plants were grown in the presence of NH_4_^+^. In plants that were not previously exposed to it, 3mM NH_4_^+^ did lead to reduced K^+^ uptake or even net K^+^ efflux. This phenomenon was particularly obvious during the first 5–8h of the assay and became more severe when the [NH_4_^+^] was raised to 10mM. Wang *et al.* (1996) also found a clear inhibitory effect of NH_4_^+^ on short-term (10min) unidirectional K^+^(Rb^+^) uptake that greatly depended on the N source and its concentration during pre-treatment. Szczerba *et al.* (2008) found that rice preferred NH_4_^+^ as N source and recorded an approximately 50% reduction in K^+^ influx when 10mM NH_4_^+^ was added to the uptake buffer.

The observed patterns of growth and K^+^ uptake in response to NH_4_^+^ were very similar between WT and KO mutants (Fig. 3). The data suggest that the inhibitory action of NH_4_^+^ is relatively short-lived and NH_4_^+^ therefore affects short-term flux assays only but not long-term growth. NH_4_^+^ is well known to depolarize the membrane (e.g. Nieves-Cordones *et al.*, 2008) which may lead to activation of outward rectifying K^+^ channels, a factor that could be responsible for the reduced uptake and K^+^ leak. In addition, our findings point to rice having a more robust high-affinity K^+^ uptake system that is largely impervious to inhibition by NH_4_^+^. This makes sense from an ecophysiological perspective: the preference of rice to absorb N in the form of NH_4_^+^ (Kronzucker *et al.*, 1998) combined with the frequent exposure to high levels of this nutrient in paddy fields (Iqbal, 2011) make it very counterproductive to depend on high-affinity K^+^ uptake that would be defunct in such conditions.

### OsAKT1 plays a role in the tolerance of rice to water stress

An important response of drought-stressed plants is the uptake of solutes such as K^+^ (e.g Marschner *et al.*, 1996; Wang *et al.* 2004; Mahouachi *et al.*, 2006) to lower the cellular water potential. Both in osmotic- (Fig. 4) and in drought-stress (Fig. 5)-grown plants the overexpression of AKT1 endowed rice with a growth advantage and a concomitant increase in root K^+^. In contrast, growth of the KO lines was less vigorous and KO roots contained less K^+^. In all, the changes in RGR correlated with AKT1 expression and root K^+^ content, suggesting that root K^+^, rather than shoot K^+^, has the main impact on growth in these conditions.

Surprisingly, OsAKT1 expression in WT rice roots is reduced by a factor 3–6 after drought treatment (GENEVESTIGATOR, https://www.genevestigator.ethz.ch/). This downregulation is not related to the drought hormone ABA, but may be caused by non-specific drought effects such as reactive oxygen species (ROS). Indeed, transcription data show AKT1 is drastically (4- to 10-fold) downregulated in response to ROS producing stresses like arsenic, anoxia and heat.

Another way in which AKT1 can impact on water homeostasis is via stomata. In Arabidopsis AKT1 is expressed at low levels in guard cell plasma membranes (Müller-Röber *et al.*, 1995; Lagarde *et al.*, 1996) and loss of function led to reduced transpiration (Nieves-Cordones *et al.*, 2012), thought to result from impaired K^+^ uptake in the guard cell and hence lower stomatal conductance. No significant difference in dry weight of the Arabidopsis plants was found, though an increase in root length was recorded for the *Atakt1* mutant in drought conditions (Nieves-Cordones *et al.* 2012). Our data, too (Fig. 4d), suggest OsAKT1 impacts on stomatal conductance during osmotic stress with KO lines showing a trend of lower conductance while conductance in OX lines was significantly higher. However, we did not find any difference in tissue water content (Supplementary Fig. 1).

### Is there a role for OsAKT1 in rice salt tolerance?

OsAKT1 shares 61% amino acid similarity with AtAKT1. OsAKT1 transcript is reduced in response to salinity, particularly in more salt-tolerant cultivars (Golldack *et al* 2003; Genevestigator), and so are AKT1-mediated currents (Fuchs, 2005). On the basis of these results and pharmacology profiles (Anil *et al.*, 2007; Wang *et al.*, 2007), it was suggested that OsAKT1 may be important in rice salt tolerance, either directly or by inducing changes in expression of other Na^+^ transporters.

We did not observe any salinity (60 or 75mM NaCl)-related growth phenotype, or significant differences in either tissue K^+^ (Supplementary Fig. 2) or Na^+^ (Fig. 6). With these levels of ambient NaCl, KO lines showed comparable shoot Na^+^ concentrations but higher levels of Na^+^ in their roots, possibly driven by the more negative membrane potential of root cells that lack AKT1 (Spalding *et al.*, 1999).

In the K^+^ deficiency condition (‘0 K’) potassium salts are replaced with sodium salts, yielding a very high external Na:K ratio (>400). It has been argued that in such circumstances inward rectifying K^+^ channels could contribute to Na^+^ uptake (Amtmann and Sanders, 1999; Amtmann and Beilby, 2010) and patch clamp recordings do indeed show residual Na^+^ current when all K^+^ is replaced with Na^+^ (Fuchs *et al.*, 2005). In addition, the pharmacology profile of ^22^Na^+^ uptake in roots and protoplasts also pointed to a role of AKT1 in Na^+^ uptake (Kader and Lindbergh, 2005; Anil *et al.*, 2007; Wang *et al.*, 2007). The 0K condition did provide some correlation between OsAKT1 expression levels and tissue Na^+^ (Fig 6b), supporting the idea that in media with a very high Na:K ratio AKT1 may directly contribute to high-affinity Na^+^ uptake.

### In conclusion

Rice is an important staple for a large part of the human population. It is often grown in areas where salinity and drought occur or where mineral nutrients such as K^+^ are deficient. AKT1 is the main inward rectifying K^+^ channel in rice roots (Golldack *et al.*, 2003; Fuchs *et al.*, 2005; Li *et al.*, 2014) and contributes considerably to K^+^ uptake at a wide range of external K^+^ levels. Our study shows that its overexpression enhances K^+^ uptake, which is beneficial in both K^+^-deficient and water-stress conditions, a phenomenon that can be exploited in future genome editing approaches.

## Supplementary data

Supplementary data are available at *JXB* online.

Figure S1. Relative water content.

Figure S2. Tissue K^+^ in salt grown rice.

Figure S3. Gene model and genotyping of KO and OX transgenic lines.

Figure S4. qRT-PCR data showing transcript levels in transgenic OX lines.

Supplementary Data

## Abbreviations:

AKT: Arabidopsis K^+^ transporter
CBL: calcineurin B-like protein
CIPK: CBL-interacting protein kinase
KO: loss of function knock out
KUE: K^+^ use efficiency
KUP/HAK/KT: K^+^ uptake permease
OX: overexpression.
