# Supplementary material for: Overexpression of the rice AKT1 potassium channel affects potassium nutrition and rice drought tolerance
Source: J Exp Bot. 2016 Mar 11;67(9):2689–98. doi: 10.1093/jxb/erw103 (PMC4861017; doi:10.1093/jxb/erw103)
Supplement: Supplementary Data [file supp_67_9_2689__index.html]

Overexpression of the rice AKT1 potassium channel affects potassium nutrition and rice drought tolerance — Overexpression of the rice AKT1 potassium channel affects potassium nutrition and rice drought tolerance — Supplementary Data 

# Overexpression of the rice AKT1 potassium channel affects potassium nutrition and rice drought tolerance

## Supplementary Data

Data files

- supplementary\_figure\_S1\_S4.pdf - Supplementary Data
