# Supplementary material for: Overexpression of the rice AKT1 potassium channel affects potassium nutrition and rice drought tolerance
Source: J Exp Bot. 2016 Mar 11;67(9):2689–98. doi: 10.1093/jxb/erw103 (PMC4861017; doi:10.1093/jxb/erw103)
Supplement: Supplementary Data [file supp_erw103_supplementary_figure_S1_S4.pdf]

Fig S1

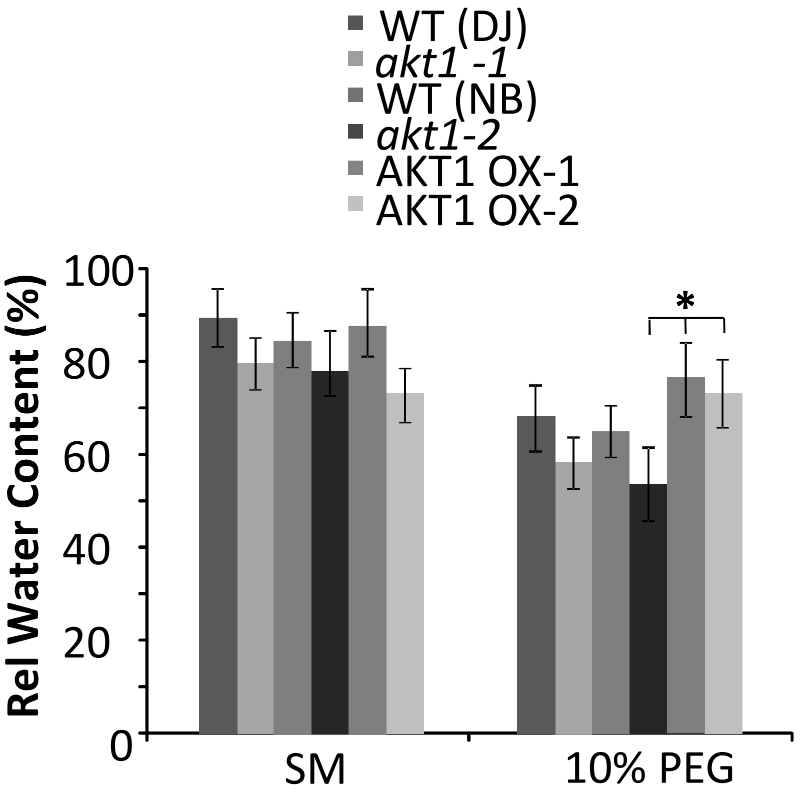

Figure S1: Relative Water Content. Relative water content was determined from the difference between FW and DW mass of 5 week old rice plants either treated in standard medium (SM) or exposed to osmotic stress in the form of 10% PEG. Data are mean values of 3-5 individual plants. Error bars denote SE, asterisk denotes significant difference between OX and KO lines ($p<0.05$).

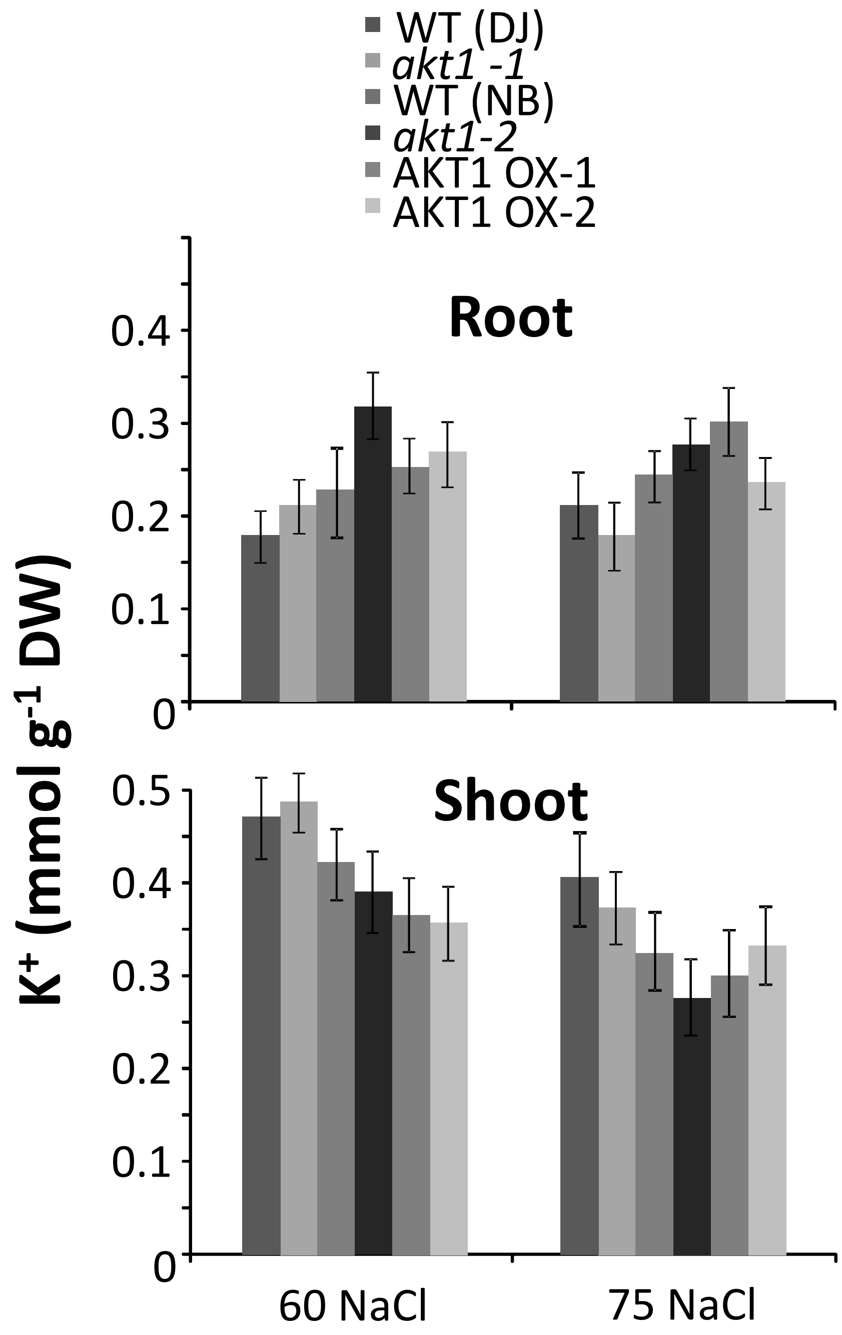

Figure S2: Tissue K^+ in salt grown rice. Root and shoot K^+ levels of rice genotypes that were grown in saline (60 or 75 mM NaCl) conditions for 2 weeks.

Fig S3

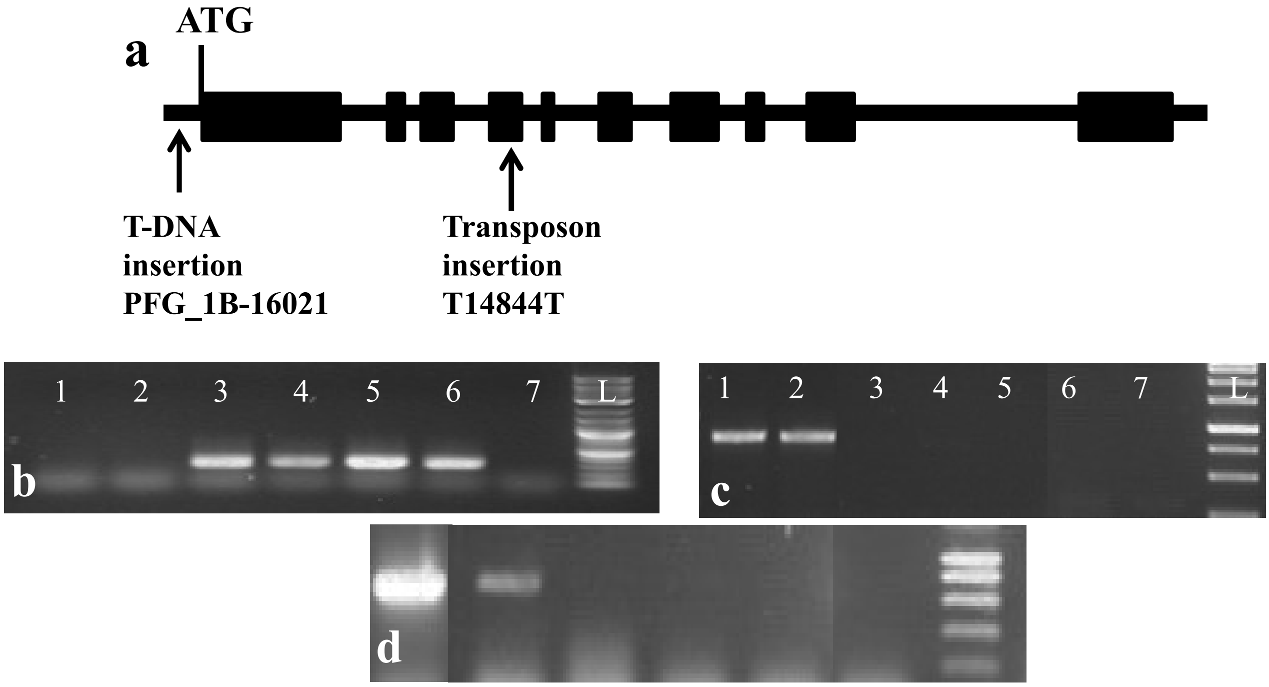

Figure S3: Gene model showing insertions in *Os-AKT1* and PCR genotyping for the *akt1* knockout line. (a) Gene model for *OsAKT1* with boxes and lines indicating the position of exons and introns respectively. The T-DNA and transposon insertion sites for *akt1-1* and *akt1-2* mutants are shown using arrows**.** The T-DNA line (*akt1-2*; PFG_1B_16021) is identical to the previously characterised *Osakt1* mutant in Li et al. (2013)**. (b)** PCR confirmation of transposon presence in *akt1-1* mutant line using transposon and *AKT1* specific primers. **(c)** RT-PCR on root extracted RNA from *akt1-1* plants using *AKT1* specific primers. No transcript was detected in 4 single *akt1-1* plants. **(d)** RT-PCR on root extracted RNA from *akt1-2* plants using AKT1 specific primers. No transcript was detected in 3 single *akt1-2* plants. Legend for (b): Lane 1 and 2 wild type; Lane 3-4 *akt1-1* and Lane 5-6 *akt1-2* insertion lines; Lane 7, water; L is the ladder. Legend for (c): Lane 1 and 2, cDNA from wild type; Lane 3-6, cDNA from *akt1-1*; Lane 7, water. Legend for (d): Lane 1 and 2, cDNA from wild type; Lane 3-5, cDNA from *akt1-2*; Lane 6, water.

Fig S4

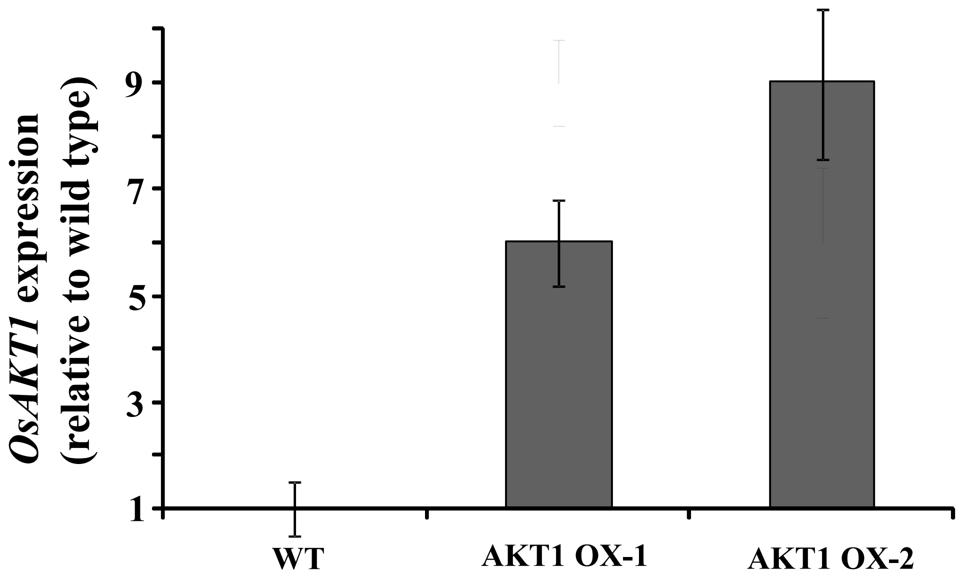

Figure S4: Analysis of the expression of *AKT1* in two transgenic overexpression lines using RT-qPCR. Three mature (5-6 week) plants of each genotype were tested for *AKT1* gene expression relative to the rice *actin-1* gene.
